# Supplementary material for: From sequence to enzyme mechanism using multi-label machine learning
Source: BMC Bioinformatics. 2014 May 19;15:150. doi: 10.1186/1471-2105-15-150 (PMC4229970; doi:10.1186/1471-2105-15-150)
Supplement: Additional file 2 — Java code of ml2db. Additional file ml2db_code.tar.gz contains the Java source code to run the multi-label machine learning experiments and save the results to database. The code’s Javadoc is included. [file 1471-2105-15-150-S2.zip › additional file 2/ml2db/ml2db/doc/main/ml2db/al/utility/neighbours/class-use/Neighbourhood.html]

Uses of Class main.ml2db.al.utility.neighbours.Neighbourhood


JavaScript is disabled on your browser.


- Overview
- Package
- Class
- Use
- Tree
- Deprecated
- Index
- Help

- Prev
- Next

- Frames
- No Frames

- All Classes

## Uses of Class main.ml2db.al.utility.neighbours.Neighbourhood

- Packages that use Neighbourhood

  | Package | Description |
  | --- | --- |
  |  |  |
  | --- | --- |
  | main.ml2db.al.utility.neighbours |  |
- - ### Uses of Neighbourhood in main.ml2db.al.utility.neighbours

    Methods in main.ml2db.al.utility.neighbours that return Neighbourhood

    | Modifier and Type | Method and Description |
    | --- | --- |
    |  |  |
    | --- | --- |
    | `Neighbourhood` | NeighbourhoodNetwork.`findBySparseAttribString(java.lang.String instanceString)` public static void main(String[] args) { int arffId = 13; String filePath = ProjectParameters.PREDICTIONS\_PATH + arffId + "\_plants" + TimeUtils.getTimeStampForFileName() + ".dot"; try { IdMultiLabelInstances plants = new IdMultiLabelInstances(arffId, ProjectParameters.getRemoteDbManagers()); NeighbourhoodNetwork network = new NeighbourhoodNetwork(plants); GraphVizPrinter .printNeighboursForGraphviz(filePath, network, false); } catch (InvalidDataFormatException e) { // TODO Auto-generated catch block e.printStackTrace(); } } |
    | `Neighbourhood` | NeighbourhoodNetwork.`findByWekaHashCode(int hashCode)` |

    Methods in main.ml2db.al.utility.neighbours that return types with arguments of type Neighbourhood

    | Modifier and Type | Method and Description |
    | --- | --- |
    |  |  |
    | --- | --- |
    | `java.util.HashMap<java.lang.String,Neighbourhood>` | NeighbourhoodNetwork.`getInstancesTree()` |
    | `java.util.Vector<Neighbourhood>` | Neighbourhood.`getIsNeededBy()` |
    | `java.util.Vector<Neighbourhood>` | Neighbourhood.`getNeededNeighbours()` |

    Methods in main.ml2db.al.utility.neighbours with parameters of type Neighbourhood

    | Modifier and Type | Method and Description |
    | --- | --- |
    |  |  |
    | --- | --- |
    | `void` | Neighbourhood.`addNeighbour(Neighbourhood neighbour)` |
    | `void` | Neighbourhood.`setIsNeededBy(Neighbourhood instance)` |

- Overview
- Package
- Class
- Use
- Tree
- Deprecated
- Index
- Help

- Prev
- Next

- Frames
- No Frames

- All Classes
